# STAT6 knockdown using multiple siRNA sequences inhibits proliferation and induces apoptosis of human colorectal and breast cancer cell lines

**DOI:** 10.1371/journal.pone.0207558

**Published:** 2019-05-10

**Authors:** Carmen Salguero-Aranda, Daniel Sancho-Mensat, Beatriz Canals-Lorente, Sabena Sultan, Ajan Reginald, Lee Chapman

**Affiliations:** Celixir, Stratford-upon-Avon, United Kingdom; Virginia Commonwealth University, UNITED STATES

## Abstract

The transcription factor STAT6 is strongly expressed in various tumours and is most highly expressed in human malignant lymphomas and pancreatic, colorectal, prostate and breast cancers. STAT6 is associated with cancer cell proliferation, an increased malignancy and poor prognosis. Thus, techniques aimed at reducing or blocking STAT6 expression may be useful in treating STAT6^high^ cancers. Among these cancers, colorectal and breast cancers represent two of the most common worldwide and their incidence is increasing every year. In 2018, colorectal and breast cancers represented 10.2% and 11.6% of all new cases of cancer diagnosed, respectively. In this study, four proprietary STAT6 specific small interfering RNA (siRNA) sequences were tested *in vitro* using the human colon adenocarcinoma cell line, HT-29, and the breast/duct carcinoma cell line, ZR-75-1. Decreases in STAT6 mRNA and protein levels were analysed to confirm the transfection was successful and STAT6 knockdown effects were measured by analysing cell proliferation and apoptosis. Results showed that 100nM siRNA concentration was the most effective and, although all individual sequences were capable of significantly inhibiting cell proliferation, STAT6 siRNA sequences 1 and 4 had the largest effects. STAT6 silencing also significantly induced apoptotic events. In conclusion, these results demonstrate that STAT6 siRNA sequences are capable of inhibiting proliferation of and inducing apoptosis of HT-29 colorectal cancer cells and ZR-75-1 breast cancer cells, halving the number of cancer cells in a short period of time. These experiments will be repeated in other STAT6^high^ cancers *in vitro*, and animal studies in immunocompromised mice have been planned using xenografts of STAT6-expressing human colorectal and breast cancer cells. The STAT6 siRNA sequences therefore represent a potential treatment for STAT6^high^ colorectal and breast cancers and a wide variety of other STAT6-expressing cancers.

## Introduction

Colorectal and breast cancers represent two of the most common cancers worldwide. Colorectal cancer (CRC) represents 10.2% of cancers worldwide, ranking third by incidence in both sexes combined (10.2%) [1]. Approximately 1.8 million new cases were diagnosed worldwide in 2018. It is, overall, the second most common cause of death by cancer globally (881,000, 9.2%), following lung cancer (18.4%), which leads cancer mortality [1]. Actual CRC treatments involve a multimodal approach based on tumour characteristics and patient-related factors. Most CRC patients with metastases are treated with a combination of chemotherapy and targeted biological drugs but, in many cases, this is only a palliative approach [2].

Breast cancer (BC) is the second most common cancer overall (2.1 million cases, 11.6%) but ranks fifth in relation to cause of death (626,000, 6.4%) because of the relatively favourable prognosis [1]. Among females, breast cancer is the most commonly diagnosed cancer and the leading cause of cancer death [1]. Newer therapeutic modalities are therefore needed to provide better treatment options for these two very common solid tumours.

The Signal Transducer and Activator of Transcription (STAT) family is formed by seven different transcription factors (STATs 1–4, 5a, 5b and 6). These proteins are important mediators in cytokine-related signalling and regulate normal cell differentiation, growth and survival [3]. However, several of the STAT genes may be considered to be oncogenes [4]. For example, STAT3 is overexpressed and active in many types of cancer, and its targeting by specific inhibitors is being investigated as a potential cancer treatment [5]. STAT6 has also been implicated in cancer. STAT6 is principally activated by two cytokines in the physiologic setting: interleukin-4 and interleukin-13 [6–10]. Once these cytokines bind to their cell surface receptors, associated Janus Kinases (Jak) are activated and phosphorylate tyrosine residues on the receptors. Cytoplasmic STAT6 docks onto the phosphorylated receptors allowing the Jaks to phosphorylate the conserved tyrosine-641 on STAT6. Once phosphorylated, two STAT6 proteins form a homodimer which translocates to the nucleus where it can directly regulate the transcription [8].

STAT6 has a well-known role in tumour immunosurveillance, immune function and lymphomagenesis but has only recently been associated with cancer progression. The STAT6 pathway has been heavily studied in animal models. STAT6-defective mice have shown immunity to mammary carcinoma [11] and also spontaneous rejection of implanted tumours [12]. In humans, high levels of STAT6 have been detected in different cancer types, including glioblastoma, lymphoma, colorectal, prostate, pancreatic, and breast cancer [13]. In addition, different studies have shown how STAT6 signalling pathway activation may be involved in the development of prostate, breast and colon carcinoma [10,14–16]. Moreover, in CRC, STAT6 is associated with increased malignancy and poor prognosis, and patients with CRC expressing STAT6 also show poor survival rates [17]. Therefore, techniques aimed at reducing STAT6 expression may be useful in treating those cancers.

Gene silencing by double-stranded (ds) RNA-mediated interference (RNAi) was first described by Craig Mello and his colleagues in 1998 [18], for which they were awarded the Nobel Prize in 2008. It is a simple and rapid method of silencing gene expression in a range of organisms by degradation of RNA into small interfering RNAs (siRNAs) that activate ribonucleases to target homologous messenger RNA (mRNA) [19]. siRNAs occur naturally from different sources (repeat-associated transcripts, viral RNAs, hairpin RNAs, *etc*) but can also be synthesized chemically and introduced into the cells. siRNAs are formed by two strands: the guide strand that assembles into the functional RNA-induced silencing complex (siRISC), which binds to the Ago protein, and the passenger strand that is discarded and degraded. The siRISC complex recognizes target RNAs by base pairing with the guide strand, leading to the silencing of the target gene through different mechanisms [20].

The ability to synthesize siRNAs has paved the way to use these molecules as potential cancer therapeutics, which can, in a targeted fashion, block gene expression of genes involved in tumour progression or maintenance of survival. They may also be used as therapeutic agents against other diseases such as viral infections and inflammatory, neurological and cardiovascular disorders. Consequently, the development of nucleotide-based biopharmaceuticals is a flourishing industry. According to recent reviews, more than 14 siRNA therapeutics have entered clinical trials in the past decade [21].

In this study, the potential effects of four proprietary STAT6 siRNA sequences, previously tested for asthma treatment [22], were examined in the human cancer cell lines HT-29 and ZR-75-1. HT-29 is a colorectal adenocarcinoma which is p53 positive and ZR-75-1 is a breast cancer line derived from an estrogen receptor-positive (ER+) ductal carcinoma [16,23]. The proliferative capacity of these cell lines depends, in part, on signalling via the STAT6 pathway. HT-29 is sensitive to standard treatments oxaliplatin, 5-fluorouracil [24] and bevacizumab (Avastin) [25] and provides a useful model for CRC therapeutic testing. BC cell line ZR-75-1 is estrogen-dependent for proliferation. It is sensitive to 5-flurouracil [26] and resistant to trastuzumab (Herceptin) [27], a standard of care agent to treat HER2 positive BC. These cell lines have both been described as STAT6^high^, so they are a good model to test the hypothesis that knocking-down STAT6 can prevent the proliferation and survival of human STAT6^high^ CRC and BC cells.

## Material and methods

### Cell culture

Human colon adenocarcinoma cell line HT-29 (Catalogue Number 91072201, ATCC HTB-38) and human breast carcinoma cell line ZR-75-1 (Catalogue Number 87012601, ATCC CRL 1500) were acquired from the European Collection of Authenticated Cell Cultures (ECCAC). In accordance with ECCAC instructions, HT-29 cells were cultured in McCoy’s 5a medium (Sigma-Aldrich) supplemented with 10% fetal bovine serum (FBS) (Sigma Aldrich), 2 mM of L-Glutamine (Sigma-Aldrich), 100 U/ml of penicillin and 100 μg/ml of streptomycin (Sigma-Aldrich) at 37°C and 5% CO_2_. Cells were passaged when 80–90% confluence was reached, and the media was changed every 2–3 days. In accordance with ECCAC instructions, ZR-75-1 cells were cultured in RPMI 1640 (Sigma-Aldrich) supplemented with 10% fetal bovine serum (FBS) (Sigma-Aldrich), 1 mM sodium pyruvate (Sigma-Aldrich), 2 mM of L-Glutamine (Sigma-Aldrich) and 100 U/ml of penicillin and 100 μg/ml of streptomycin (Sigma-Aldrich) at 37°C and 5% CO_2_. Cells were passaged when 70–80% confluence was reached, and the media was changed every 2–3 days.

### siRNA transfection

HT-29 and ZR-75-1 cells were seeded in 6-well and 12-well plates at a concentration of 15,000 cells/cm^2^. In all experiments, as ZR-75-1 cells grow more slowly than HT-29 cells, the transfection was carried out at later time points for ZR-75-1 cells than for HT-29 cells. HT-29 cells were transfected 24 hours post-culture with the four siRNA sequences at different final concentrations using DharmaFECT Transfection Reagent 1 (Dharmacon) or jetPEI (Polyplus) in antibiotic-free media, following the manufacturer’s instructions. ZR-75-1 cells were transfected 3 days post-culture using DharmaFECT Transfection Reagent 2 (Dharmacon). jetPEI transfection was developed using a ratio of reagent:siRNA (μL:μg) of 2:1 for HT-29 cells. The sense strands of the duplex STAT6 siRNAs were: Sequence 1 (STAT6.1): 5' GCAGGAAGAACUCAAGUUUUU 3’, Sequence 2 (STAT6.2): 5' ACAGUACGUUACUAGCCUUUU 3', Sequence 3 (STAT6.3): 5' GAAUCAGUCAACGUGUUGUUU 3’, Sequence 4 (STAT6.4): 5' AGCACUGGAGAAAUCAUCAUU 3'. Sequential transfections in HT-29 cells were developed using STAT6.1 and STAT6.4 at 100 nM. Non-targeting siRNA (Dharmacon) was used as negative control at 10 to 200 nM, depending on the assay. Media was not changed until the first 48 hours and antibiotic-free media was always used.

### RNA isolation, reverse transcription and q-PCR

24 hours (HT-29 cells) or 3 days (ZR-75-1 cells) post-transfection, total RNA was isolated using a microRNA Isolation Kit (Qiagen) according to the manufacturer’s instructions directly from the plate. mRNA was then quantified by Nanodrop 1000-ND and 1 μg of RNA was transcribed into complementary DNA (cDNA) using SuperScript III Reverse Transcriptase (Invitrogen) following the manufacturer’s instructions. The DNA primers used were Human STAT6 Forward: 5' CTTTCCGGAGCCACTACAAG 3’ and reverse 5' AGGAAGTGGTTGGTCCCTTT 3'; Human GAPDH Forward: 5' -TGCACCACCAACTGCTTAGC 3' and reverse 5 ' GGCATGGACTGTGGTCATGAG 3'. GAPDH expression was measured as endogenous control. The quantitative PCR (qPCR) was performed in a 7900HT Real-time PCR system (ThermoFisher). The program cycle was: initial denaturation for 5 min at 95°C, followed by 40 cycles of 15 sec at 95°C and 60 sec at 60°C. A melt curve was added at the end of the process. The data was analysed by Delta-Delta Ct method.

### STAT6 protein detection

2, 5 and 7 days (HT-29 cells) or 4 and 7 days (ZR-75-1 cells) post-transfection, cells were harvested and fixed and permeabilized with Cell Signalling Buffer Set A (Miltenyi) according to the manufacturer’s instructions. In brief, cells were fixed for 10 min at room temperature (RT) with the Inside Fix Buffer and permeabilized for 30 min at 4°C with the Permeabilization Buffer pre-cooled at -20°C. Cells were washed twice with PBS/0.5%BSA (Bovine Serum Albumin) and stained with anti-STAT6 APC conjugated antibody (Miltenyi Biotec, 130-104-030) (20 μl/10^6^ cells) for 30 min in the dark at 4°C. Antibody isotype REA Control (I)-APC (Miltenyi, 130-104-615) was used as a negative control. The stained cells were washed once and resuspended finally in 400 μL of PBS/0.5%BSA, before analysing them by flow cytometry (FACSCalibur, BD). Data was analysed using FlowJo software (FlowJo, BD).

### Cell proliferation

HT-29 cells were grown for 5 and 7 days post-transfection to analyse individual transfection, and 14 days for sequential transfection assay, while the ZR-75-1 cells were grown for 4 and 7 days after transfection. 2 or 3 days post-transfection the media was replaced, and every 2 days, fresh antibiotic-free media was added. Cell number was used as a measure for cell proliferation. Total and dead cells were counted using a NucleoCounter NC-100 (Chemometec) and live cells were then calculated.

### Apoptosis analysis

For both cell lines, HT-29 and ZR-75-1, cells were harvested 7 days after transfection and stained with anti-Annexin V FITC-conjugated antibody (BD Bioscience, 556420) at 20 μl/1 X 10^6^ cells in Binding Buffer 1X (BD Bioscience), for 15 min RT protected from light. Cells were finally resuspended in 400 μl of Binding Buffer 1X and 100 μl of propidium iodide (PI) solution (250 nM) (Sigma-Aldrich) was added to the cells and incubated for 1 min before analysing with the flow cytometer (FACSCalibur, BD). Data was analysed using FlowJo software (FlowJo, BD).

### Statistical analysis

The statistical analysis was carried out using PRISM software, by Student’s t-distribution of unpaired data, two-tailed, and 95% level of confidence. Values were compared to the non-targeting (NT) siRNA transfected cells. Significant values: *(p-value <0.05), **(p-value<0.01), ***(P<0.001), ****(P-value<0.0001).

## Results

### STAT6 siRNA optimal dose and best sequences

**Fig 1:**
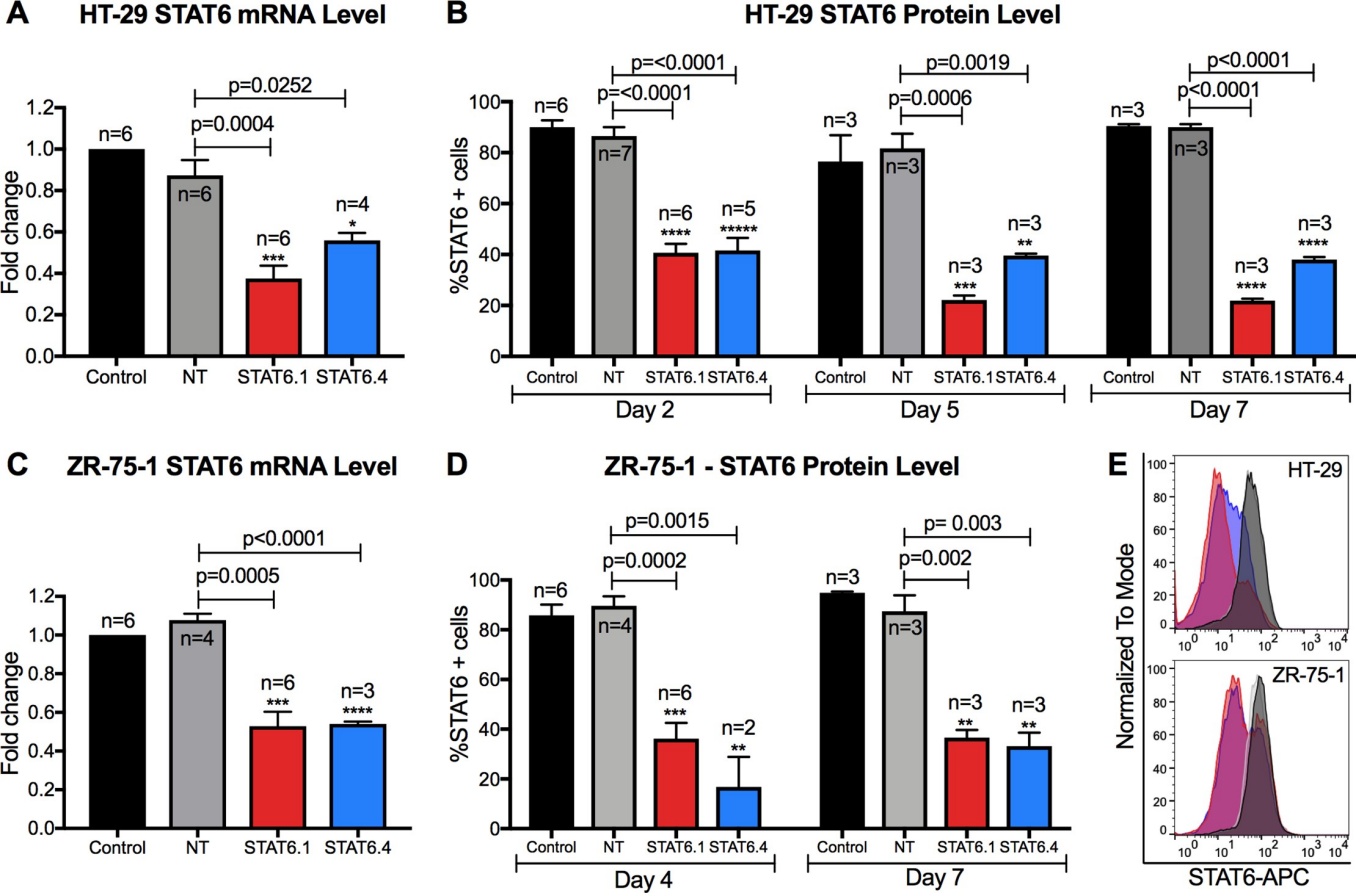
STAT6 siRNA sequences 1 and 4 significantly block STAT6 expression. (A) Measure of STAT6 mRNA level in HT-29 cells 24 hours post-transfection. The graph represents the mean ± SEM of multiple independent experiments (n) obtained by real-time PCR. Results were analysed by ΔΔCt method for relative quantifications. The fold change is represented by the Y axis, and values are normalized to control cells. (B) STAT6 protein level analysis at 2, 5 and 7 days post-transfection in HT-29 cells. The graph represents the mean of the percentage of STAT6 positive cells ± SEM of multiple independent experiments (n) obtained by flow cytometry. (C) Measure of STAT6 mRNA level in ZR-75-1 cells 3 days post-transfection. The graph represents the mean ± SEM of multiple independent experiments (n) obtained by real-time PCR. Results were analysed by ΔΔCt method for relative quantifications. The fold change is represented by the Y axis, and values are normalized to control cells. (D) STAT6 protein level analysis after 4 and 7 days of transfection in ZR-75-1 cells. The graph represents the mean of the percentage of STAT6 positive cells ± SEM of multiple independent experiments (n) obtained by flow cytometry. E) Representative histograms (Control: back; NT: grey; STAT6.1: red; STAT6.4: blue) of STAT6 protein analysis at 7 days post-transfection by flow cytometry in HT-29 cells (top) and ZR-75-1 (bottom) cells. STAT6 siRNA sequences and non-targeting siRNA were used at 100 nM as the final concentration. Control cells were non-transfected cells and STAT6 siRNA sequences 1 and 4 and non-targeting siRNA are denoted as STAT6.1, STAT6.4 and NT, respectively. The number of independent experiments (n) is set out in the Fig.

In order to test the four proprietary STAT6 siRNA sequences’ efficiency, the first step was to determine the optimal dose in HT-29 cells. Ascending concentrations of STAT6: 10, 25, 50, 100 and 200 nM were tested for each STAT6 siRNA sequence and both STAT6 mRNA and protein levels were measured. Results illustrated that all four sequences worked efficiently at silencing STAT6 expression (S1 Fig). All conditions tested showed significant changes versus cells treated with non-targeting siRNA (NT), with the exception of 10 and 25 nM of STAT6 sequence 2 (STAT6.2) and 10 nM of STAT6 sequence 3 (STAT6.3) at mRNA level. Regarding the expression of the STAT6 protein, all conditions showed statistically significant changes, with 100 and 200 nM being the most effective, achieving an average of more than 60% knockdown for the four sequences. No significant changes were observed between 100 and 200 nM (S1 Fig). For this reason, 100 nM was established as the STAT6 siRNA optimal dose and this concentration was used for the remaining assays. To determine the effects of STAT6 siRNA on HT-29 cell proliferation, cells were transfected with 100 nM of each siRNA sequence and counted at different time points. Results showed that STAT6.2 and STAT6.3 reduced the number of live cells after 7 days of transfection by approximately 20–30%, while STAT6 siRNA sequences 1 (STAT6.1) and 4 (STAT6.4) achieved a reduction of approximately 50% (S2 Fig). The reduction of the total number of cells when STAT6.1 and STAT6.4 were used was also appreciable under the inverted microscope (S2C Fig). To demonstrate the efficacy of STAT6.1 and STAT6.4 when used at 100 nM, more biological replicates were developed, and these clearly demonstrated that STAT6 expression was reduced by approximately 50% at mRNA level in transfected HT-29 cells (Fig 1A). To test how long transient siRNA knockdown lasts, STAT6 protein expression was analysed by flow cytometer at 2, 5 and 7 days post-transfection. The data revealed that STAT6 fluorescence was decreased in STAT6.1 and STAT6.4 transfected cells after 2 days (>50%), and the transient knockdown was maintained up to 7 days post-transfection. Both siRNA sequences resulted in more than 50% knockdown at day 5 and 7 post-transfection (Fig 1B). In the same way as in HT-29 cells, the STAT6 mRNA levels were analysed in transfected ZR-75-1 cells, and results showed that STAT6.1 and STAT6.4 significantly decreased STAT6 mRNA expression by 50% (Fig 1C). Furthermore, STAT6 protein expression was measured in transfected ZR-75-1 cells at 4 and 7 days post-transfection. The results showed that STAT6 expression was decreased in ZR-75-1 cells transfected with 100 nM of STAT6.1 and STAT6.4 by 60 and 80%, respectively, when compared with the NT cells at day 4. The transient siRNA knockdown in ZR-75-1 cells was also confirmed to last 7 days after transfection (Fig 1D). The flow cytometry histograms clearly showed that the decrease in STAT6 expression was maintained for 7 days in STAT6.1 and STAT6.4 HT-29 and ZR-75-1 transfected cells (Fig 1E). Thereby, 100 nM and STAT6.1 and STAT6.4 were established to be the optimal dose and sequences, respectively, and they were used for the subsequent experiments.

### STAT6 siRNA sequences 1 and 4 significantly reduce cell proliferation

**Fig 2:**
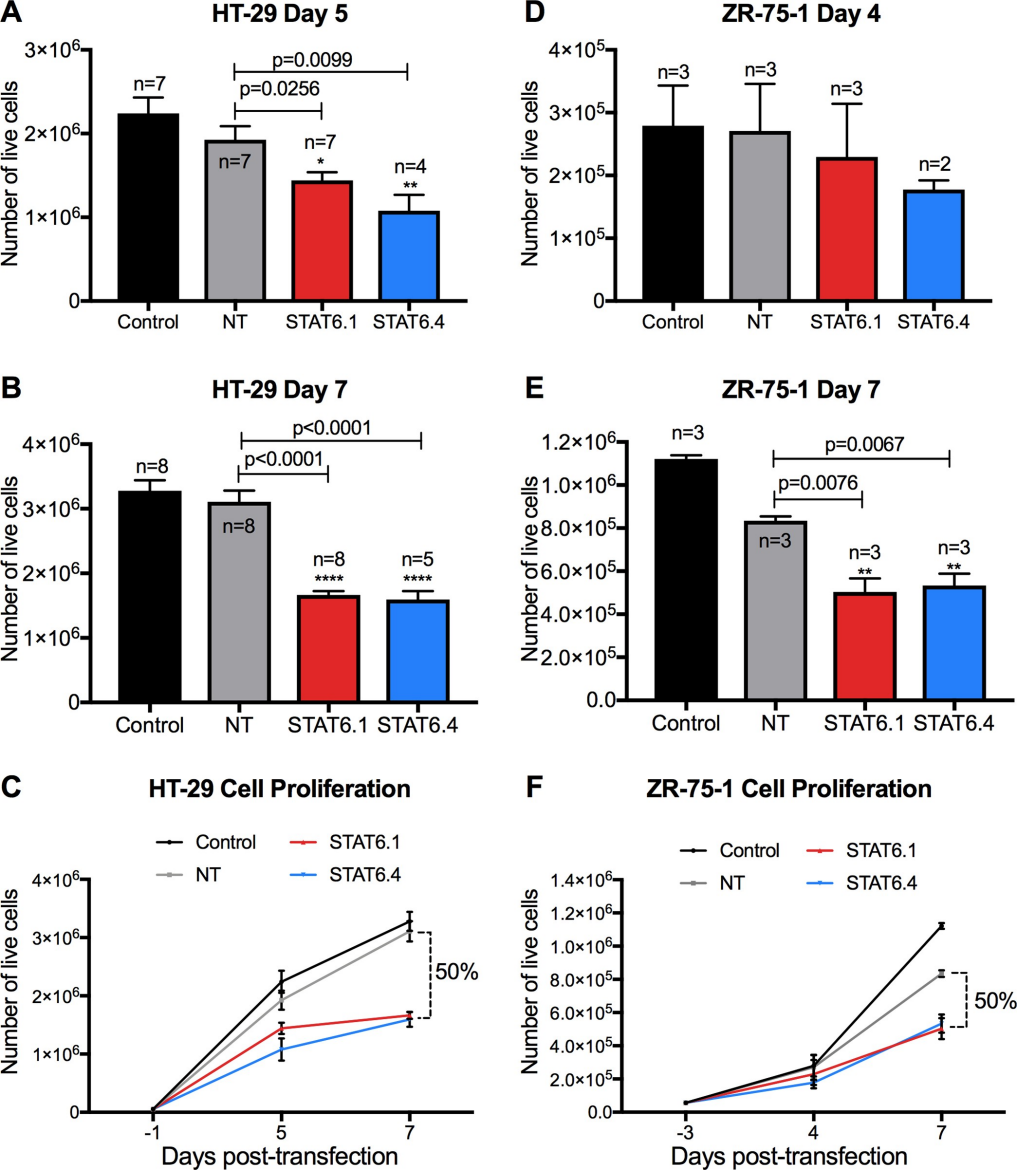
STAT6 siRNA sequences 1 and 4 significantly reduce cell proliferation. (A and B) Number of live HT-29 cells measured at 5 and 7 days post-transfection, respectively. The graphs represent the mean ± SEM of multiple independent experiments (n). (C) The graph illustrates how HT-29 cells grew over time and represents the mean ± SEM of the independent experiments (n) shown in A and B. (D and E) Number of live ZR-75-1 cells measured at 4 and 7 days post-transfection, respectively. The graphs represent the mean ± SEM of multiple independent experiments (n). (F) The graph illustrates how ZR-75-1 cells grew over time and represents the mean ± SEM of the multiple independent experiments (n) shown in D and E. The number of live cells was calculated as detailed in the material and methods using NucleoCounter NC-100. The percentage of reduction of the number of live cells was calculated by comparison between the mean of NT *vs*. the mean of the transfection with STAT6 siRNA sequences. STAT6 and non-targeting (NT) siRNA sequences were used at 100 nM as the final concentration. Non-transfected cells served as negative control and STAT6 siRNA sequences 1 and 4 and non-targeting siRNA are denoted as STAT6.1, STAT6.4 and NT, respectively. The number of independent experiments (n) is set out in the Fig.

Once the optimal dose and optimal STAT6 siRNA sequences were determined, their effects on the cell proliferation of the HT-29 and ZR-75-1 cells were analysed. The results in HT-29 cells showed that NT transfected cells presented a similar growth pattern to non-transfected cells (control), while STAT6.1 and STAT6.4 siRNA treatments significantly decreased HT-29 cell proliferation, reducing by half the number of cancer cells at 7 days post-transfection in comparison with cells transfected with NT (Fig 2A, 2B and 2C). As can be seen from Fig 2E, the DharmaFECT transfection reagent 2 and NT siRNA reduced ZR-75-1 cell proliferation compared with untransfected cells (control). This probably results from some level of toxicity of the reagent 2. Nevertheless, STAT6.1 and STAT6.4 significantly reduced the proliferation rate of the cells because the number of live cells was reduced by half at 7 days post-transfection (but not at 4 days post-transfection) when compared with NT transfected cells (Fig 2E and 2F). These experiments demonstrated that the STAT6 siRNA sequences 1 and 4 are capable of significantly reducing the number of HT-29 and ZR-75-1 cells *in vitro* in a short period of time.

### STAT6 siRNA sequences induce apoptotic events

**Fig 3:**
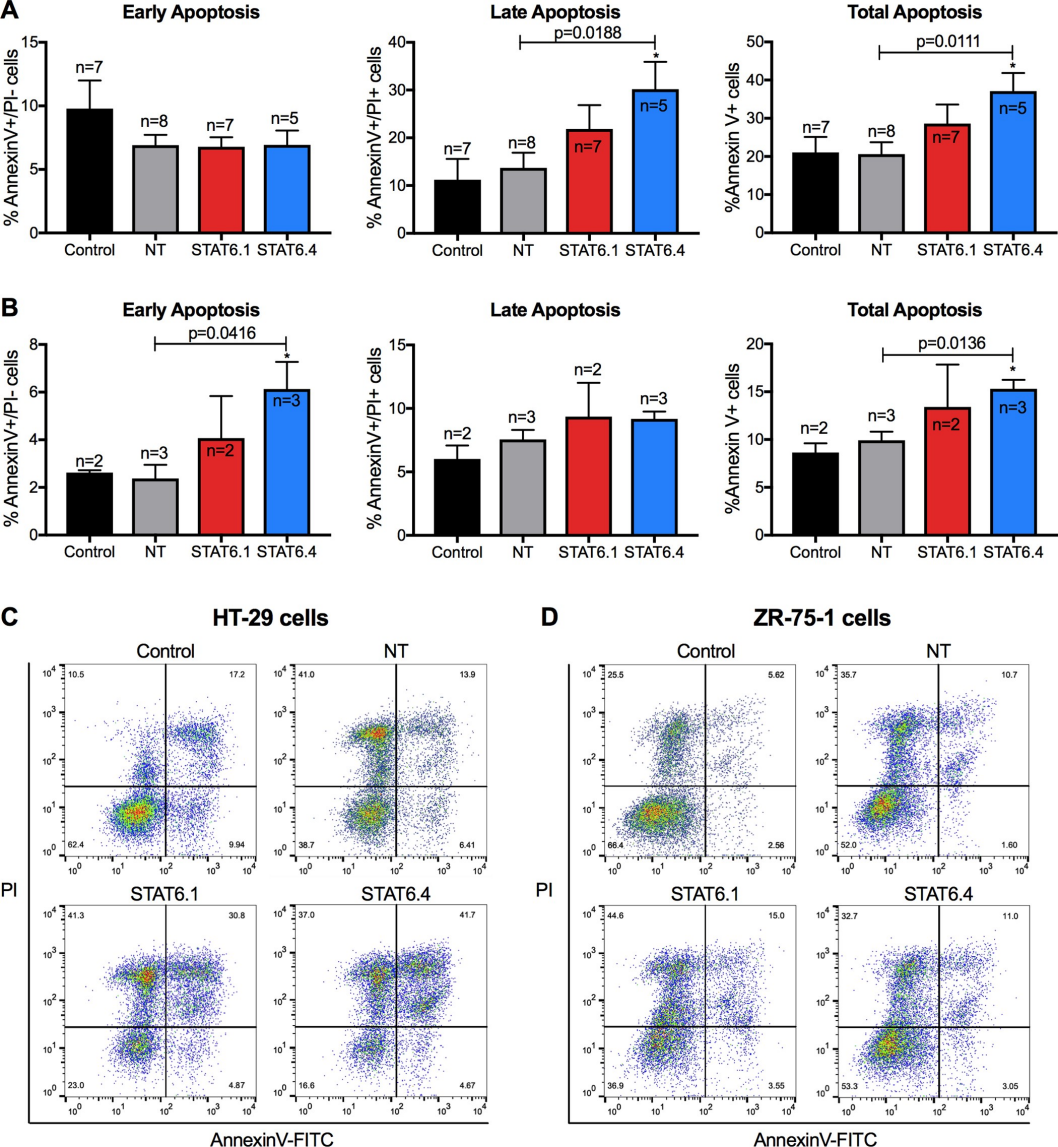
STAT6 siRNA sequences 1 and 4 induce apoptosis. (A) Apoptosis analysis in HT-29 cells. (B) Apoptosis analysis in ZR-75-1 cells. In both cases, the graphs represent from left to right: early (Annexin V^+^/PI^-^), late (Annexin V^+^/PI^+^) and total (Annexin V^+^) apoptosis. The graphs represent the mean ± SEM of multiple independent experiments (n) obtained by flow cytometry. (C) Representative flow cytometry plots in HT-29 cells. (D) Representative flow cytometry plots in ZR-75-1 cells. The X axes represent Annexin V and the Y axes represent PI fluorescence intensity. Quadrants were set according to cells independently stained with Annexin V or PI. Apoptosis was studied at 7 days post-transfection in both cell lines. Data were analysed with Flowjo Software. STAT6 siRNA sequences and a non-targeting siRNA sequence were used at 100 nM as the final concentration. Non-transfected cells served as control cells and STAT6 siRNA sequences 1 and 4 and non-targeting siRNA are denoted as STAT6.1, STAT6.4 and NT, respectively. The number of independent experiments (n) is set out in the Fig.

STAT6.1 and STAT6.4 were clearly shown to significantly reduce the number of live HT-29 and ZR-75-1 cells over time, so their effects on apoptosis were also tested. After 7 days post-transfection, HT-29 and ZR-75-1 cells were harvested, counterstained with Annexin V and PI and analysed by flow cytometry. The data showed that while early apoptosis (Annexin V^+^/ PI^-^) was not affected, late (Annexin V^+^/PI^+^) and total (Annexin V^+^) apoptosis were increased in STAT6.4 transfected HT-29 cells (Fig 3A and 3C). Conversely, early apoptosis was significantly increased in STAT6.4 transfected ZR-75-1 cells. In the same way, total apoptosis was also induced in ZR-75-1 cells (Fig 3B and 3D). This discrepancy in the apoptosis stage induced by the STAT6 siRNAs in both cell lines may be due to the different growth rates of the cell lines. As ZR-75-1 cells demonstrate a longer doubling time than HT-29 cells, the detection of late apoptosis in ZR-75-1 cells could require a longer cell culture period than was tested.

### STAT6 siRNA sequential transfection works at maintaining a reduced number of cancer cells over time

**Fig 4:**
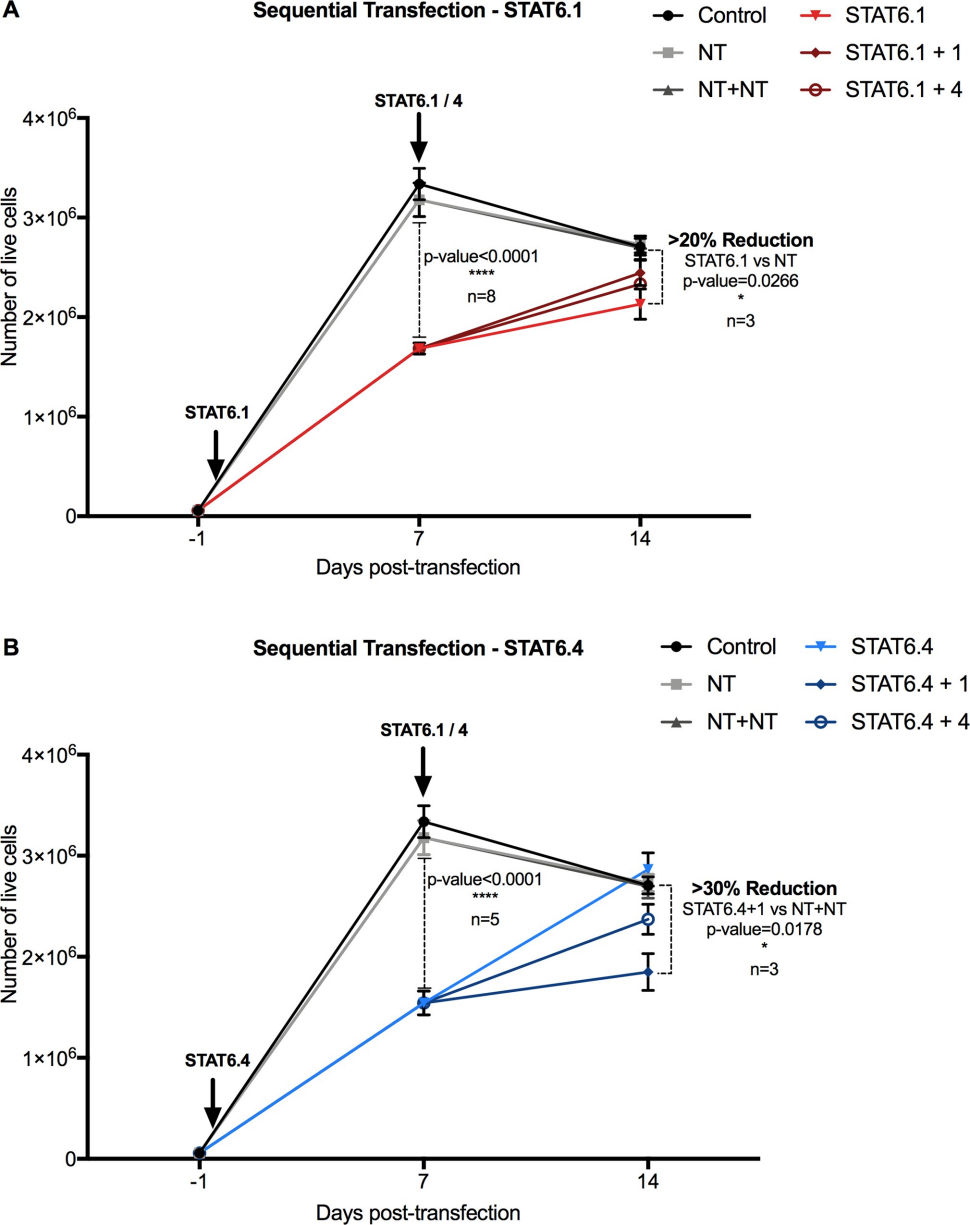
STAT6 siRNA sequential transfection is effective in maintaining a reduced number of HT-29 cells. STAT6 siRNA transfection was carried out at day 1 of cell culture with (A) STAT6.1 and (B) STAT6.4 at 100 nM. A second transfection was carried out in both cases with STAT6.1 and STAT6.4 at the same concentration 7 days after the first transfection. The graphs represent the number of live cells over time measured at day 7 and 14 days post-first transfection counted using NucleoCounter NC-100 as detailed in the material and methods section. The mean ± SEM of 3 independent experiments is represented in each graph. Control cells were non-transfected cells and STAT6 siRNA sequences 1 and 4 and non-targeting siRNA are denoted as STAT6.1, STAT6.4 and NT, respectively. The percentage of reduction of the number of live cells was calculated by comparison between the mean of NT *vs*. the mean of the individual transfection with STAT6 siRNA sequences, and sequential transfection with NT (NT+NT) *vs*. sequential transfection with STAT6.1 and STAT6.4.

It is clear from these results that STAT6.1 and STAT6.4 at 100 nM significantly reduced the number of live HT-29 and ZR-75-1 cells cultured for up to 7 days post-transfection. Further experiments in HT-29 cells were conducted to see if the effects of the STAT6 siRNA sequences could be extended. Sequential transfection using 100nM STAT6.1 and STAT6.4 at each transfection was developed. The first transfection was carried out as before, and 7 days later, a second transfection was performed using the same STAT6 siRNA sequence (STAT6.1 or STAT6.4) or the other STAT6 siRNA sequence. Overall, four different sequential transfections were carried out: STAT6.1+1, STAT6.1+4, STAT6.4+1 and STAT6.4+4. A sequential transfection with the NT sequence and individual transfections with NT, STAT6.1 and STAT6.4 were also carried at the same time as controls. Then, HT-29 cells were cultured for a total of 14 days after the first transfection. The results showed a similar grow pattern between the control cells (non-transfected) and NT transfected cells (Fig 4).

Exponential growth was expected between days 7 and 14 in control and NT cells, similar to what was observed between days 0 and 7. However, the rate of cell growth decreased between days 7 and 14 (Fig 4) and this was probably due to the limited size of the culture surface (3.8 cm2), which resulted in the cultures approaching confluence. Nonetheless, significant differences were still observable in the STAT6 transfected cells. STAT6.1 resulted in more than 20% reduction in the number of live cells when compared with the NT counterpart (Fig 4A), while the sequential combination of STAT6.4 and STAT6.1 siRNAs (STAT6.4+1) achieved more than 30% reduction when compared with the NT+NT treatment (Fig 4B). These data confirmed that sequential injections in animal models could be effective extending the effects of the STAT6 siRNA sequences.

### JetPEI transfection reagent works for STAT6 siRNA treatment *in vitro*

**Fig 5:**
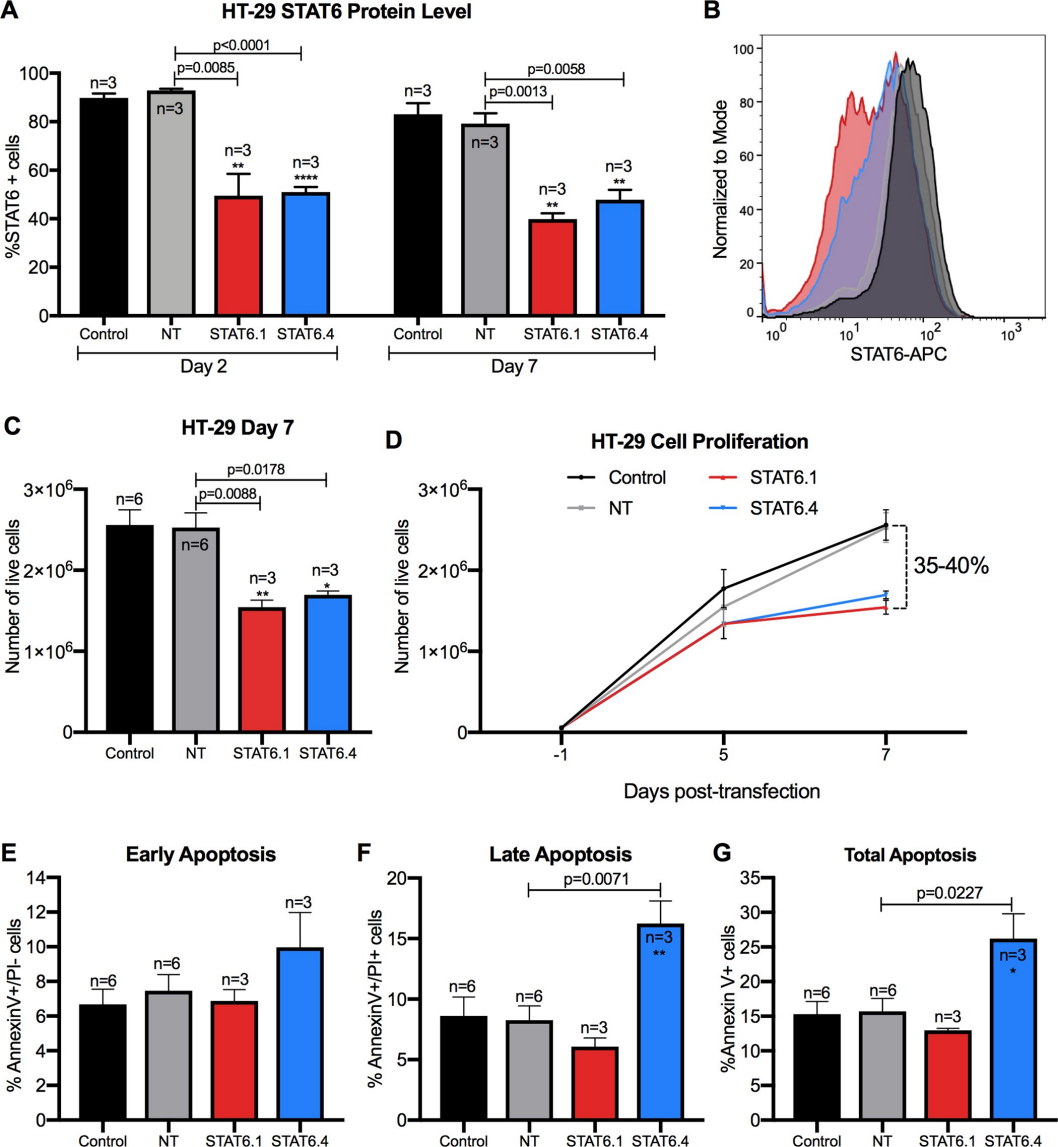
JetPEI transfection reagent works for transfecting efficiently STAT6 siRNAs *in vitro*. (A) STAT6 expression at protein level in HT-29 cells at day 2 and 7 post-transfection measured by flow cytometry. The graph represents the mean ± SEM of multiple independent experiments (n). Data was analysed using Flowjo Software. The percentage of STAT6 positive cells is represented on the Y axis. (B) Representative histograms (Control: back; NT: grey; STAT6.1: red; STAT6.4: blue) of STAT6 protein analysis at 7 days post-transfection by flow cytometry in HT-29 cells. (C) Number of live HT-29 cells measured at day 7 post-transfection by NucleoCounter NC100. The graph represents the mean ± SEM of multiple independent experiments (n). (D) The graph illustrates how HT-29 cells grew over time and represents the mean ± SEM of the independent number of experiments shown in C. (E, F and G) The graphs show early (E) (Annexin V^+^/PI^-^), late (F) (Annexin V^+^/PI^+^) and total (G) (Annexin V^+^) apoptosis, and represent the mean ± SEM of multiple independent experiments (n) obtained by flow cytometry. Apoptosis was studied at 7 days post-transfection. Data were analysed with Flowjo Software. STAT6 siRNA sequences and a non-targeting siRNA sequence were used at 100 nM as the final concentration. Non-transfected cells served as control cells and STAT6 siRNA sequences 1 and 4 and non-targeting siRNA are denoted as STAT6.1, STAT6.4 and NT, respectively. The number of independent experiments (n) is set out in the Fig.

The previous experiments were conducted using DharmaFECT, a lipid-based transfection reagent that provides efficient and reliable transfection at low concentrations with minimal cellular toxicity, but its use has not been tested *in vivo*. Therefore, once it was established that STAT6.1 and STAT6.4 individually at 100 nM had significant effects on cell proliferation and apoptosis of HT-29 cells, the efficacy of these STAT6 siRNA sequences was tested using a transfection reagent with proven efficacy *in vivo*. JetPEI reagent is a linear polyethylenimine derivative, free of components of animal origin, providing a highly effective and reproducible gene delivery to adherent and suspension cells and with a similar composition to *in vivo*-jetPEI, which is widely use in *in vivo* studies. Results using jetPEI showed that STAT6 protein expression was reduced by more than 40% when both STAT6.1 and STAT6.4 were used. Moreover, it was again confirmed that the STAT6 knockdown was maintained for 7 days post-transfection (Fig 5A and 5B). The next step was to analyse if the effects of STAT6 siRNAs on HT-29 cell proliferation and apoptosis were reproducible when jetPEI was used. The results showed that the number of HT-29 live cells were significantly decreased after 7 days post-transfection, obtaining 35 and 40% reductions of the number of live cells with STAT6.1 and STAT6.4, respectively (Fig 5C and 5D). The apoptosis analysis also proved the effectiveness of jetPEI. The treatment with STAT6.4 showed an increased number of early (Annexin V^+^/PI^-^) (Fig 5E), late (Annexin V^+^/PI^+^) (Fig 5F) and total (Annexin V^+^) (Fig 5G) apoptotic events. These results show that the jetPEI transfection reagent could be a successful option for future animal studies.

## Discussion

CRC and BC represent two of the most common cancers worldwide and their incidence is increasing every year [1]. More than 2 millions of new cases of BC were detected worldwide in 2018, while more than 1.7 millions of CRC were diagnosed, representing 11.6% and 10.2% of all new cases of cancer detected, respectively [1]. The 5-year relative survival rate for patients with stage IIIC and IV CRC is approximately 53% and 11% respectively [28]. According to the World Cancer Research Fund International, since 2008, worldwide BC incidence has increased by more than 20%. Mortality has increased by 14% [1]. Thus, exploring the underlying mechanism of CRC and BC cells and finding new treatment targets are essential for improving the survival rate of CRC and BC patients.

Several studies have shown that STAT6 plays an important role in the progression and proliferation of several different types of cancer. Barbara C Merk *et al*. demonstrated in 2011 [29] that STAT6 acts to enhance cell proliferation and invasion in glioblastoma, which may explain why up-regulation of STAT6 correlates with shorter survival times in glioma patients. A study in 2007 showed that the actions of STAT6 in lung cancer were directly involved in COX-2 expression [30]. A more recent study suggests that miR-135b functions as a tumour suppressor, affecting the metastatic ability of prostate cells by targeting STAT6, and STAT6 knockdown resulted in reduced cell metastasis. Furthermore, the expression of miR-135b was observed to be associated with the pathological T stages and levels of total and free PSA in patients with prostate cancer [31]. It has been also shown that the inhibition of the STAT6 pathway in tumour-associated macrophages (TAMs) is a vital therapeutic approach to attenuate tumour growth and metastatic niche formation in breast cancer [32]. In the same way, Yan D. *et al*. determined that cytokine-activated STAT3 and STAT6 cooperate in macrophages to promote a secretory phenotype that enhances tumour progression in a cathepsin-dependent manner [33]. STAT6 is also associated with an increased malignancy and a poor prognosis in CRC patients [17]. Moreover, it has been demonstrated that the IL-13/IL-13Rα1/STAT6/ZEB1 pathway plays a critical role in promoting aggressiveness of CRC [34]. For these reasons STAT6 was chosen in this study and the reported results suggest that the STAT6 siRNA sequences, especially STAT6.1 and STAT6.4, have the potential to treat CRC and BC that highly express this transcription factor.

Interestingly, it has been demonstrated that the STAT6^null^ breast cancer cell line (BT-20) exhibits an increased apoptosis as compared with STAT6^high^ ZR-75-1 cell line on day 6 in spontaneous culturing without any treatment [16]. In addition, in comparison with BT-20 cells, the ZR-75-1 cell line appeared to have more constitutively expressed genes known to regulate resistance to apoptosis, which might be relevant to the observed resistance to apoptosis in ZR-75-1 cells [16]. Moreover, STAT6^null^ colon cancer cells (Caco-2) showed an increased spontaneous apoptosis and, at the same time, decreased metastatic capability, compared with HT-29 cells. HT-29 cells also showed resistance to apoptosis and vigorous metastatic capability [10].

This study is not the first one that investigated the effects of STAT6 knockdown in HT-29 cells. Zhang MS *et al*. showed in 2006 that STAT6-specific short hairpin RNAs (shRNAs) inhibit proliferation and induce apoptosis in CRC HT-29 cells [35]. They analysed the expression of total STAT6 and phosphorylated STAT6 protein by semiquantitative RT-PCR, obtaining a significant reduction of the STAT6 expression. HT-29 cell viability was also tested 72 hours post-transfection, and the results showed a greatly decreased viability. Apoptosis analysis by flow cytometry indicated that STAT6 shRNAs induced significant early apoptotic events (Annexin V^+^/ PI^-^ cells). In contrast in this study, STAT6.4 also induced late apoptosis (Annexin V^+^/ PI^+^). This may be due to the fact that the apoptosis assay was analyzed after 7 days post-transfection, which would allow the STAT6 pathway to complete its action mechanism, or that the STAT6 siRNA sequences are more powerful at inducing the apoptosis of the cancer cells. In this study, the effects of STAT6 siRNA over a longer period of time (7 and 14 days) were also investigated and this provided new data regarding the effects of STAT6 silencing on cell proliferation and apoptosis in cancer cells. Moreover, Zhang *et al*. used shRNA, which is expressed after nuclear delivery of an shRNA-expressing plasmid DNA (pDNA), and the duration of shRNA expression depends on the use of viral or non-viral vectors. Conversely, the delivery of siRNAs as in this study avoids the barrier of the nuclear membrane as it acts in the cytosol [36].

siRNAs offer additional advantages over shRNAs. Pre-designed siRNA duplexes are available from various sources or can be custom designed. Furthermore, siRNAs are easy to modify to increase their stability without altering their structure and efficiency and can be conjugated with fluorophores for *in vivo* tracking. In addition to this, the amount of exogenous nucleic acid introduced into the cells is much lower, as siRNAs consist of only duplexes of 19 nucleotide pairs and no insertion vector is required, thus reducing probable side effects. It is for these and other reasons why siRNAs are becoming a popular tool for cancer therapy. To date, approximately 20 clinical trials have been initiated using siRNA-based therapeutics. However, several barriers still exist to achieving effective and controlled *in vivo* delivery and these limits the use of siRNAs in the clinic. In post-intravenous injection, the siRNA complex must navigate the circulatory system of the body while avoiding kidney filtration, uptake by phagocytes, aggregation with serum proteins and enzymatic degradation by endogenous nucleases [37,38]. The current siRNA delivery systems for cancer therapy mainly include chemical modifications of siRNA, lipid-based, polymer-based, and conjugate siRNA delivery systems, as well as co-delivery of siRNA and anticancer drugs, and inorganic nanoparticles [39]. These modifications help to address the problems associated with naked siRNA delivery and effectively introduce the siRNA inside the target cells.

In this study, two transfection reagents were tested, DharmaFECT and jetPEI. The former is a lipid-based formulation and the latter is a linear polyethylenimine (PEI) derivative. Both of these reagents effectively delivered the STAT6 siRNAs into the cells because STAT6 expression was significantly knocked down in both cases. Nevertheless, jetPEI, unlike DharmaFECT, has been successfully tested in several animal studies and is known to form stable complexes with the nucleic acid, protecting it from degradation. Moreover, good manufacturing practice (GMP) grade *in vivo*-jetPEI is being used in several ongoing preclinical studies and phase I and II clinical trials. Thus, this makes jetPEI an excellent candidate for future animal and clinical studies using the STAT6 siRNA sequences tested in this study.

In conclusion, the proprietary STAT6 siRNAs used in this study silenced STAT6 expression, reduced the number of live cells and induced apoptosis in the STAT6^high^ CRC and BC cell lines, HT-29 and ZR-75-1, respectively. Animal studies using immunocompromised mice with human CRC and BC xenografts are currently being planned. These will allow the determination of the *in vivo* effectiveness of the STAT6 siRNA sequences. These findings, together with the observation of constitutive and high STAT6 expression in many human malignant cancers, suggest that the STAT6 siRNAs can be used as successful anticancer therapies against human STAT6^high^ cancers.

## Supporting information

S1 Fig Optimal dose of STAT6 siRNA sequences in HT-29 cell line. (A) STAT6 mRNA level measure at 24 hours post-transfection. The graphs represent the mean ± SEM of 3 independent experiments. Values were obtained by real-time PCR and results were analysed by ΔΔCt method for relative quantifications. The fold change is represented on the Y axes, and values are normalized to control cells. (B) STAT6 protein level analysis. The graphs represent the mean of the percentage of STAT6 positive cells ± SEM of 2 independent experiments obtained by flow cytometry. The percentage of STAT6 positive cells is represented on the Y axes. STAT6 siRNAs and non-targeting siRNA were used at 10, 25, 50, 100 and 200 nM as the final concentrations. Control cells were non-transfected cells and STAT6 siRNA sequences 1, 2, 3 and 4 and non-targeting siRNA are denoted as STAT6.1, STAT6.2, STAT6.3 and STAT6.4 and NT, respectively.
(TIFF)

S2 Fig Measure of HT-29 cell proliferation using 100 nM STAT6 siRNA sequences 1 to 4. Number of live cells measured at day (A) 2, (B) 5 and (C) 7 post-transfection. The graphs represent the mean ± SEM of 2 independent experiments. (D) The graph shows how cells grew over time and represents the mean ± SEM of the independent experiments shown in A, B and C. The percentage of reduction of the number of live cells is calculated by comparison between the mean of NT *vs*. the mean of STAT6 siRNAs. The number of live cells was calculated as detailed in the material and methods section using NucleoCounter NC-100. STAT6 siRNAs and non-targeting siRNA were used at 100 nM as final concentration. (E) Inverted microscope images taken at day 7 after transfection. Scale bar = 300 μm. Control cells were non-transfected cells and STAT6 siRNA sequences 1, 2, 3 and 4 and non-targeting siRNA are denoted as STAT6.1, STAT6.2, STAT6.3 and STAT6.4 and NT, respectively.
(TIFF)
