# Expression of Concern: STAT6 knockdown using multiple siRNA sequences inhibits proliferation and induces apoptosis of human colorectal and breast cancer cell lines

**DOI:** 10.1371/journal.pone.0246415

**Published:** 2021-01-28

**Authors:** Carmen Salguero-Aranda, Daniel Sancho-Mensat, Beatriz Canals-Lorente, Sabena Sultan, Ajan Reginald, Lee Chapman

After this article [1] was published, questions were raised as to the compliance of this article with PLOS’ Materials Sharing and Competing Interests policies, about the ownership and scope of patents that apply to the siRNAs used in the article, and about aspects of the statistical analyses, results, and conclusions.

- The article refers to the STAT6 siRNA sequences as “proprietary” as they are covered by patents owned by SIRNA Ltd., a daughter company of Celixir PLC. The patents cover Europe, Australia, Canada, New Zealand, US, South Africa, and cover the sequences for any use including development and/or sale of research reagents (EP1725658, AU2005217200, CA2599524, NZ549915, US7,566,700 and ZA2006/07471 which are all national/regional phases of the Walker and Hopkin patent in ref. 22). The authors provided documentation to support these statements regarding ownership of the relevant patents.
- The sequences of the siRNAs are provided in the Materials and Methods section of [1]. The authors have clarified that they will grant interested researchers permissions to use the siRNAs and/or siRNA sequences for non-commercial purposes such as academic research. Researchers with commercial affiliations may request collaboration for use of the siRNAs and/or siRNA sequences under commercial license agreements. Patent rights will be asserted in cases where the sequences or reagents based on the sequences are used for commercial activities, such as selling reagents.

The Competing Interests statement in [1] was incomplete and is updated to:

LC and SS were formerly Directors of Cell Therapy Ltd (CTL), which at that time was owned by Celixir PLC. Before assuming this role, LC, as a contracted patent attorney, assisted CTL in filing a UK patent application related to the siRNAs studied in this article. AR currently is a Director of CTL, Director and CEO of Celixir PLC, and Director of SIRNA Ltd. (the company which owns the siRNA patents reported in this article). The commercial affiliation with Celixir PLC does not alter our adherence to PLOS ONE policies on sharing materials and data. It should be noted that patents for the four siRNA sequences used in the study are owned by SIRNA Ltd., but the siRNA sequences will be made available to others under a license to be negotiated with Celixir PLC and without restrictions upon publication of the work using the sequences. All potential licenses will require the signing of a Material Transfer Agreement (MTA). Celixir welcomes requests from non-commercial institutions to use the siRNAs and/or the siRNA sequences for non-commercial purposes, e.g. for academic research. Whether or not a fee is charged to academics will depend on the circumstances. Celixir is open to collaboration from researchers with commercial affiliations under a normal commercial licence agreement

The Data Availability Statement is updated to:

The underlying data to support results reported in this article are available from Figshare: https://figshare.com/projects/STAT6_knockdown_using_multiple_siRNA_sequences_inhibits_proliferation_and_induces_apoptosis_of_human_colorectal_and_breast_cancer_cell_lines/64889

The sample size for STAT6.4 is misreported in Fig 1A. The figure reports n = 4 but the correct sample size is n = 3.

**Fig 1:**
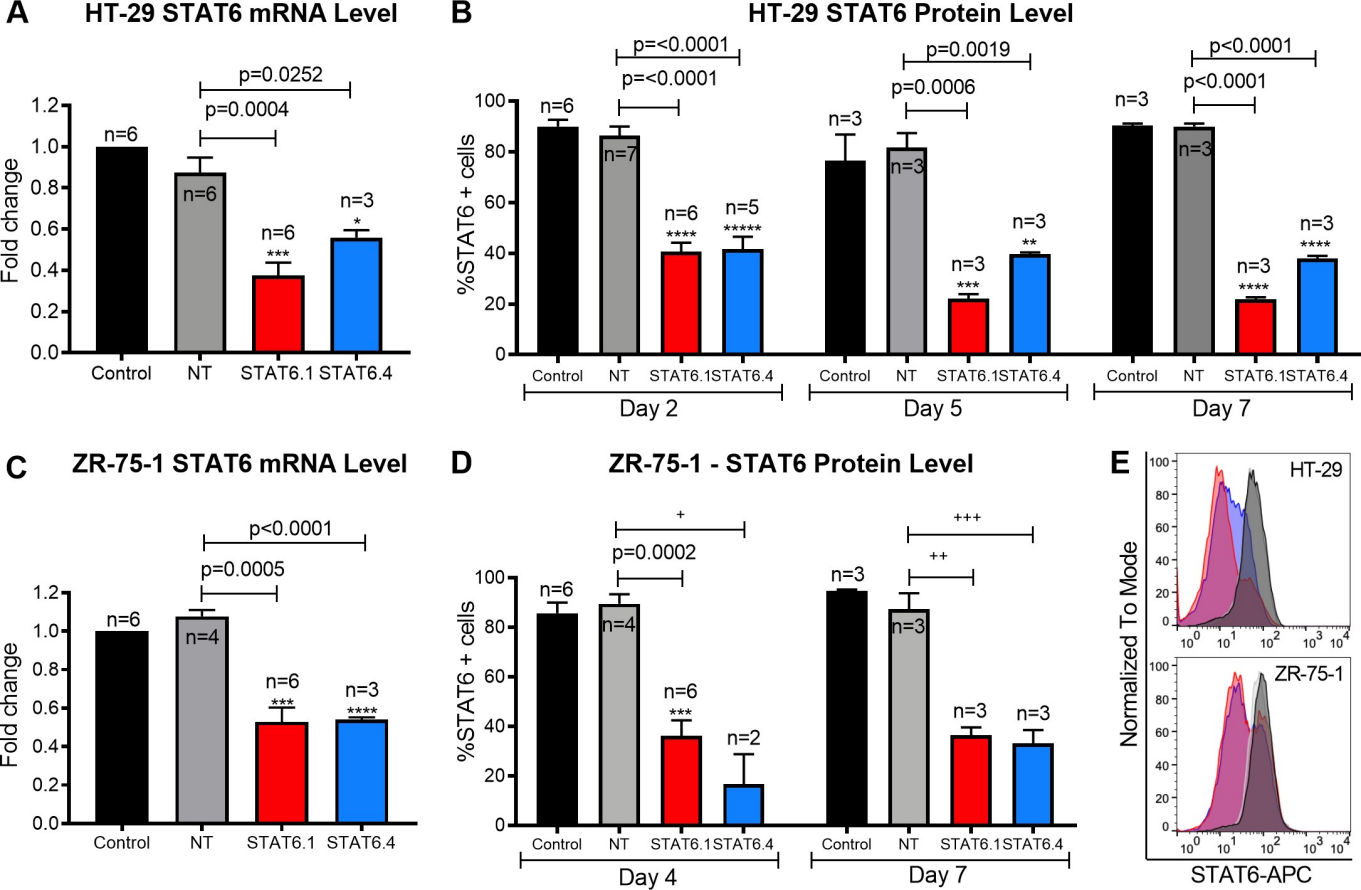
STAT6 siRNA sequences 1 and 4 significantly block STAT6 expression. (A) Measure of STAT6 mRNA level in HT-29 cells 24 hours post-transfection. The graph represents the mean ± SEM of multiple independent experiments (n) obtained by real-time PCR. Results were analysed by ΔΔCt method for relative quantifications. The fold change is represented by the Y axis, and values are normalized to control cells. (B) STAT6 protein level analysis at 2, 5 and 7 days post- transfection in HT-29 cells. The graph represents the mean of the percentage of STAT6 positive cells ± SEM of multiple independent experiments (n) obtained by flow cytometry. (C) Measure of STAT6 mRNA level in ZR-75-1 cells 3 days post-transfection. The graph represents the mean ± SEM of multiple independent experiments (n) obtained by real-time PCR. Results were analysed by ΔΔCt method for relative quantifications. The fold change is represented by the Y axis, and values are normalized to control cells. (D) STAT6 protein level analysis after 4 and 7 days of transfection in ZR-75-1 cells. The graph represents the mean of the percentage of STAT6 positive cells ± SEM of multiple independent experiments (n) obtained by flow cytometry. +: n is too small to test normality and equality of variances. The p-value for a t-test is 0.0015, but p = 0.0801 for unequal variances (Welch´s correction) and p = 0.1333 for non-parametric tests (unpaired two-tailed Mann Whitney test). The significance of the result must be considered with caution. ++: The p-value for a t-test is 0.02, but, for non-parametric samples, p = 0.1 for an unpaired two-tailed Mann Whitney test. The significance of the result must be considered with caution. +++: The p-value for a t-test is 0.003 but, for non-parametric samples, p = 0.1 for an unpaired two-tailed Mann Whitney test. The significance of the result must be considered with caution. E) Representative histograms (Control: back; NT: grey; STAT6.1: red; STAT6.4: blue) of STAT6 protein analysis at 7 days post-transfection by flow cytometry in HT-29 cells (top) and ZR-75-1 (bottom) cells. STAT6 siRNA sequences and non-targeting siRNA were used at 100 nM as the final concentration. Control cells were non-transfected cells and STAT6 siRNA sequences 1 and 4 and non-targeting siRNA are denoted as STAT6.1, STAT6.4 and NT, respectively. The number of independent experiments (n) is set out in the Fig.

Questions were raised post-publication as to whether the results and conclusions are adequately supported given that the experiments were conducted only in a single cell line per cancer type, lacked some controls, and sample sizes were low (as few as two replicates for some experiments). Questions were also raised about the validity of the statistical analyses reported in the article.

The authors provide the following clarifications:

As reported in the Methods, the article reports results conducted in a single colon adenocarcinoma cell line (HT-29) and a single breast carcinoma cell line (ZR-75-1). Experiments reported in Figs 4, 5, S1 and S2 were conducted only in HT-29 cells. After the article was published the authors conducted preliminary replication experiments using the lung cancer cell line A549. In these experiments, the siRNA sequences reduced STAT6 expression but did not significantly affect cell numbers or apoptosis (S1 File). Further experiments using additional cell lines or in vivo models are needed to examine the generalizability of the article’s results.
Each full experiment reported in the article included at least two biological replicates, where each replicate corresponds to cells harvested at different passage numbers. The same number of starting cells and same experimental conditions were used for each biological replicate. For real time PCR experiments (Fig 1A and 1C) technical replicates were included, in which each biological sample was loaded into three different wells for triplicate PCR reactions. Within the dataset the different groups within each experiment do not all have the same number of replicates. Some data were excluded due to contamination and because not all groups were included in optimization experiments which contributed to replicates included in final analyses.
For the statistical analyses reported in [1], the authors assumed that the data (including for datasets where n = 2) followed a Gaussian distribution and both populations had the same Standard Deviation (SD). F-test was used to compare variances and most cases showed a p-value of >0.05, so similar variances between groups was assumed. Results with only two replicates were only considered statistically significant when the values were dramatically different when compared with NT (e.g. p-value = 0.0015 in Fig 1D). In the other cases (i.e. Fig 2D and Fig 3B), the Student’s t-test provided p values higher than 0.05 and so the results were not significant (and no significance was claimed in the published article). In this context, the published article only reports the reduced expression of STAT6 protein in ZR-75-1 cells after 4 days following STAT6.4 siRNA transfection (Fig 1D) as being considered significant when only 2 replicates were done. This is consistent with the function of the siRNA (to reduce STAT6 protein expression) and also consistent with the data in Fig 1C showing a significant reduction in the amount of STAT6 mRNA in the same cell type for an experiment in which n = 3.

A statistical reviewer reviewed the article and underlying dataset and raised concerns about the assumptions made about normality and equal variances. The article did not report evidence to support these assumptions or information about how the authors assayed for normality and equal variances. The authors provided the following updates in response:

The Shapiro-Wilk test by individual groups was applied to test normality of the sample population when n ≥3. Among all datasets with n ≥3, only values of Fig 1D, Day 7 for NT, and S1 Fig panel A, STAT6.3, 25 and 200 nM, did not pass the Shapiro-Wilk test. For these data, t tests may not be reliable; an unpaired two-tailed Mann Whitney test (a nonparametric version of the independent samples t-test) was performed and data became non-significant (p-value was 0.1 for all the cases) (S1 Table). All other data with n ≥3 passed the Shapiro-Wilk test and were assumed to be normal; t tests were used to analyze these data.
F-test was used to test the equality of variances (S2 File). When F-test reported non-significant differences the Student’s t-distribution of unpaired data and two-tailed, with 95% confidence, was performed. However, when variances were unequal, a two-tailed unpaired t test with Welch's correction was done as well.
Cases for which variance was unequal are reported in S2 Table with this notice; the significant/non-significant results for these cases were the same whether data were analyzed using the t test or the Welch’s correction test.
When n was < 3, normality and variance equality tests could not be applied (n too low) and normality of the data and equal variances were assumed, and a t test was performed. However, the S3 Table shows the statistical differences when parametric and nonparametric tests and equal and unequal variance test were applied.
Results of statistical analyses for Figs 2 and 3 are provided in S4 and S5 Tables, respectively.

Updated versions of Fig 1 and S1 Fig. are provided here to address reporting errors and updates regarding the statistical analysis results. As noted in the updated figure legends, several results are not statistically significant when analyzed using different methods and assumptions.

The reviewer advised that statistical tests to assess normality and equality of variance would be expected to have very low power given the small sample sizes in this study, and as such may yield false negative results. This has implications for whether appropriate methods were used to analyze the results of the experiments. Some experimental results are notably different when data are analyzed using methods that are suitable for data with normal vs. non-normal distributions or equal versus unequal variances. In light of these issues, the reviewer advised that results should be interpreted with caution.

A member of *PLOS ONE*’s Editorial Board likewise advised that the limited dataset remains of concern and some broad statements in the article are not adequately supported by the results.

In addition, the conclusions reported in [1] overstate what is supported by the reported data. The final statement in the Abstract and the final statement in the Discussion are hereby replaced with the following:

The results of these studies suggest that inhibition of STAT6 signaling by siRNA may provide a useful strategy for therapy of colorectal and breast cancers expressing high levels of STAT6.

Finally, reference 22 is incomplete in [1]. The correct reference is:

22. Walker W and Hopkin J (2005). *Materials and methods for treatment of allergic disease*. WO 2005/083083 A2. [patent] International application published under the Patent Cooperation Treaty (PCT). Available from: https://patentimages.storage.googleapis.com/d3/1e/37/de26e889cea8e8/WO2005083083A2.pdf.

The *PLOS ONE* Editors issue this Expression of Concern as we consider that the concerns about the sample size and statistical analyses are not fully resolved, and questions remain as to the reliability and robustness of the results.

## Supporting information

S1 File Supplemental A549 data provided post-publication. (XLSX)

S2 File All data (including Shapiro-Will and F tests). (XLSX)

S1 Fig Optimal dose of STAT6 siRNA sequences in HT-29 cell line. (A) STAT6 mRNA level measure at 24 hours post-transfection. The graphs represent the mean ± SEM of 3 inde- pendent experiments. Values were obtained by real-time PCR and results were analysed by ΔΔCt method for relative quantifications. The fold change is represented on the Y axes, and values are normalized to control cells. +: The p-value for a t-test is 0.0106, but, for non-parametric samples, p = 0.1 for an unpaired two-tailed Mann Whitney test. The significance of the result must be considered with caution. ++: The p-value for a t-test is 0.0056, but, for non-parametric samples, p = 0.1 for an unpaired two-tailed Mann Whitney test. The significance of the result must be considered with caution. (B) STAT6 protein level analysis. The graphs represent the mean of the percentage of STAT6 positive cells ± SEM of 2 independent experiments obtained by flow cytometry. The percentage of STAT6 positive cells is represented on the Y axes. STAT6 siRNAs and non-targeting siRNA were used at 10, 25, 50, 100 and 200 nM as the final concentrations. Control cells were non-transfected cells and STAT6 siRNA sequences 1, 2, 3 and 4 and non-targeting siRNA are denoted as STAT6.1, STAT6.2, STAT6.3 and STAT6.4 and NT, respectively.
(TIF)

S1 Table List of data that did not pass normality test (Shapiro-Wilk p-value<0.05). (DOCX)

S2 Table List of data that did not pass equality of variance test (F test p-value<0.05). Comparison of statistical significances analyzed with equal (t test) and unequal (Welch´s correction) distribution variance tests.
(DOCX)

S3 Table Resume of cases where n<3. (DOCX)

S4 Table Statistical analysis of Fig 2. (DOCX)

S5 Table Statistical analysis of Fig 3. (DOCX)
