# Supplementary figures and images for: STAT6 knockdown using multiple siRNA sequences inhibits proliferation and induces apoptosis of human colorectal and breast cancer cell lines
Source: PLoS One. 2019 May 10;14(5):e0207558. doi: 10.1371/journal.pone.0207558 (PMC6510441; doi:10.1371/journal.pone.0207558)

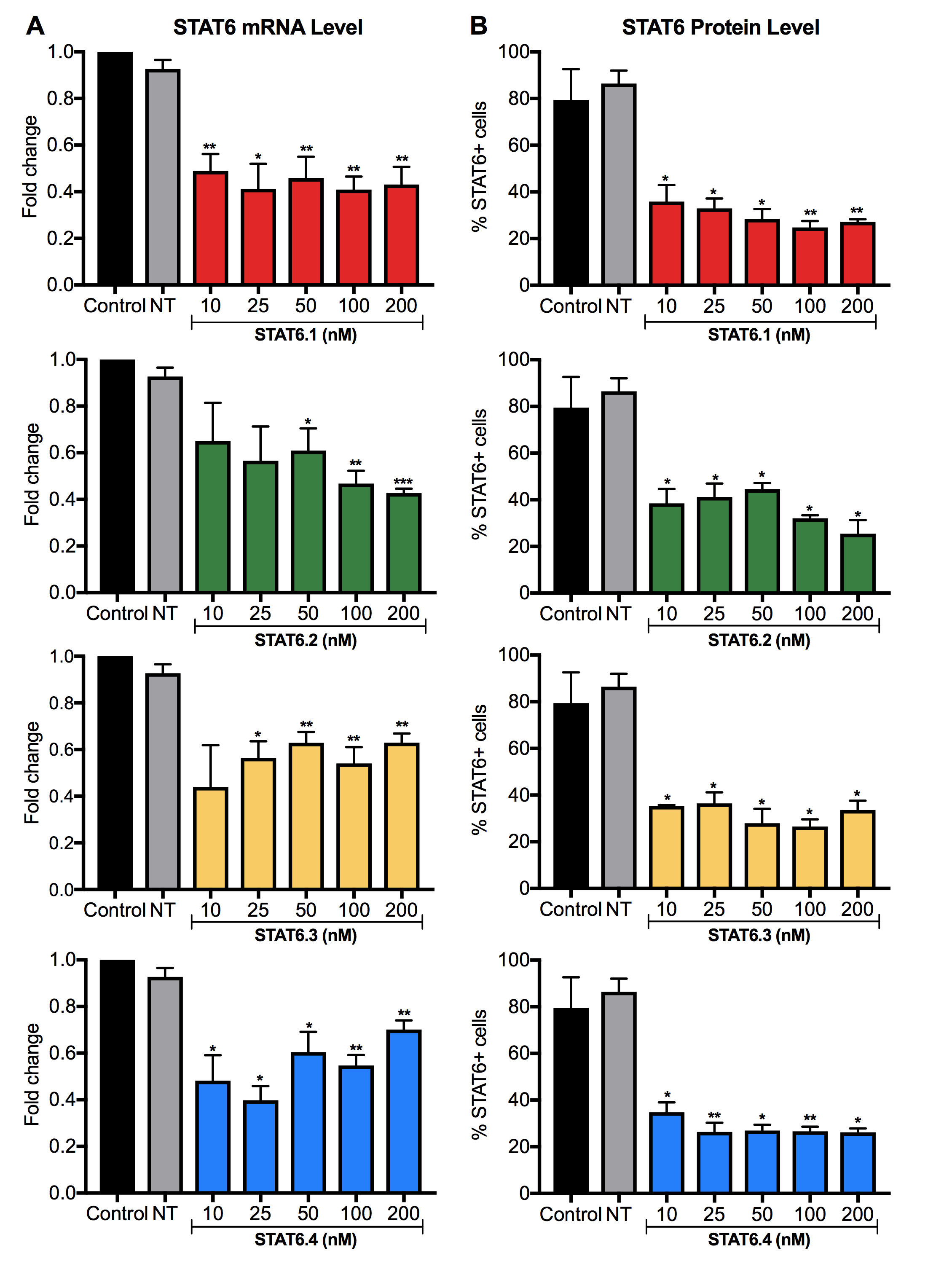

Supplement: S1 Fig — (A) STAT6 mRNA level measure at 24 hours post-transfection. The graphs represent the mean ± SEM of 3 independent experiments. Values were obtained by real-time PCR and results were analysed by ΔΔCt method for relative quantifications. The fold change is represented on the Y axes, and values are normalized to control cells. (B) STAT6 protein level analysis. The graphs represent the mean of the percentage of STAT6 positive cells ± SEM of 2 independent experiments obtained by flow cytometry. The percentage of STAT6 positive cells is represented on the Y axes. STAT6 siRNAs and non-targeting siRNA were used at 10, 25, 50, 100 and 200 nM as the final concentrations. Control cells were non-transfected cells and STAT6 siRNA sequences 1, 2, 3 and 4 and non-targeting siRNA are denoted as STAT6.1, STAT6.2, STAT6.3 and STAT6.4 and NT, respectively. (TIFF) [file pone.0207558.s001.tiff]

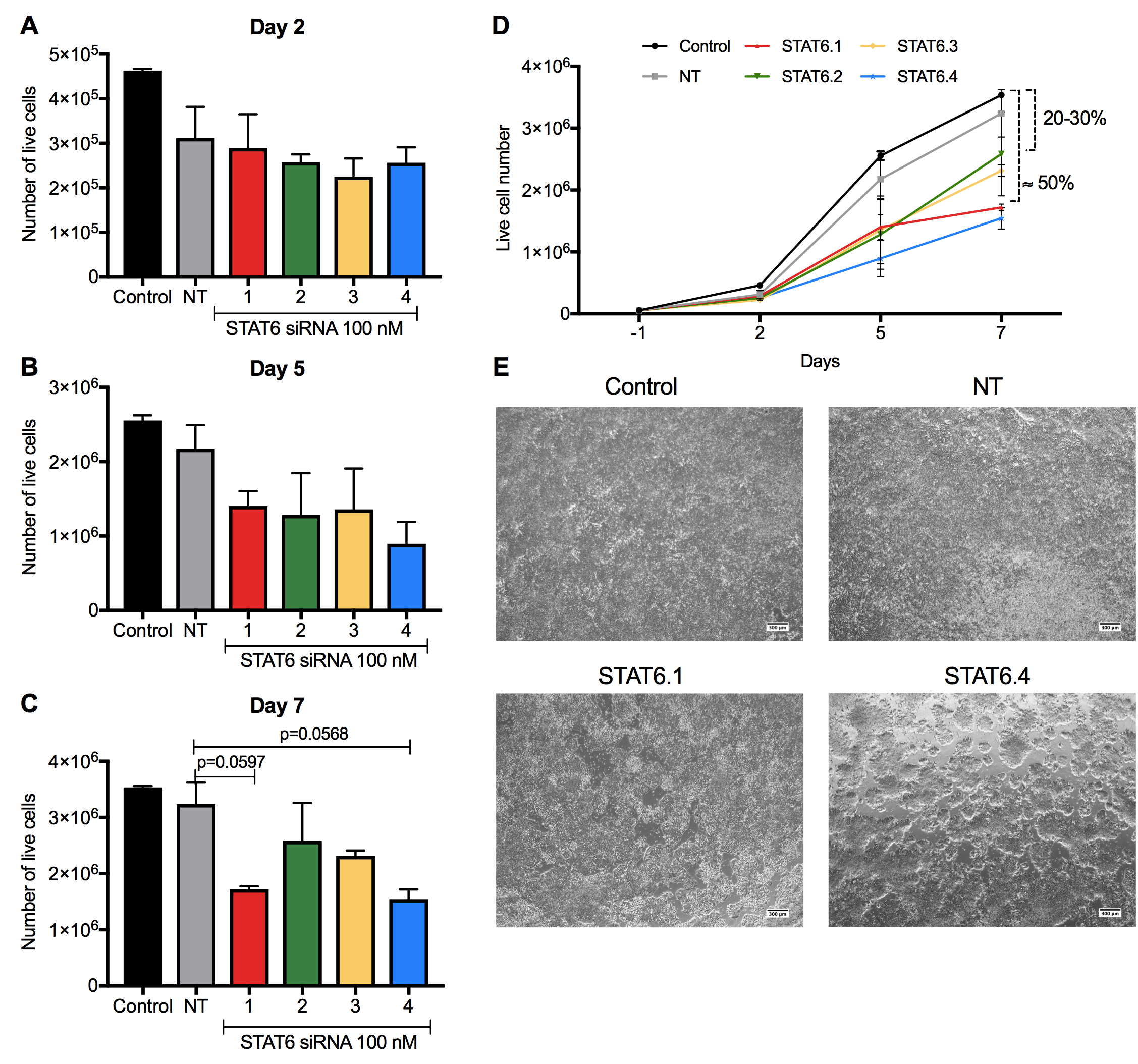

Supplement: S2 Fig — Number of live cells measured at day (A) 2, (B) 5 and (C) 7 post-transfection. The graphs represent the mean ± SEM of 2 independent experiments. (D) The graph shows how cells grew over time and represents the mean ± SEM of the independent experiments shown in A, B and C. The percentage of reduction of the number of live cells is calculated by comparison between the mean of NT vs. the mean of STAT6 siRNAs. The number of live cells was calculated as detailed in the material and methods section using NucleoCounter NC-100. STAT6 siRNAs and non-targeting siRNA were used at 100 nM as final concentration. (E) Inverted microscope images taken at day 7 after transfection. Scale bar = 300 μm. Control cells were non-transfected cells and STAT6 siRNA sequences 1, 2, 3 and 4 and non-targeting siRNA are denoted as STAT6.1, STAT6.2, STAT6.3 and STAT6.4 and NT, respectively. (TIFF) [file pone.0207558.s002.tiff]
