# Supplementary figures and images for: Expression of Concern: STAT6 knockdown using multiple siRNA sequences inhibits proliferation and induces apoptosis of human colorectal and breast cancer cell lines
Source: PLoS One. 2021 Jan 28;16(1):e0246415. doi: 10.1371/journal.pone.0246415 (PMC7842988; doi:10.1371/journal.pone.0246415)

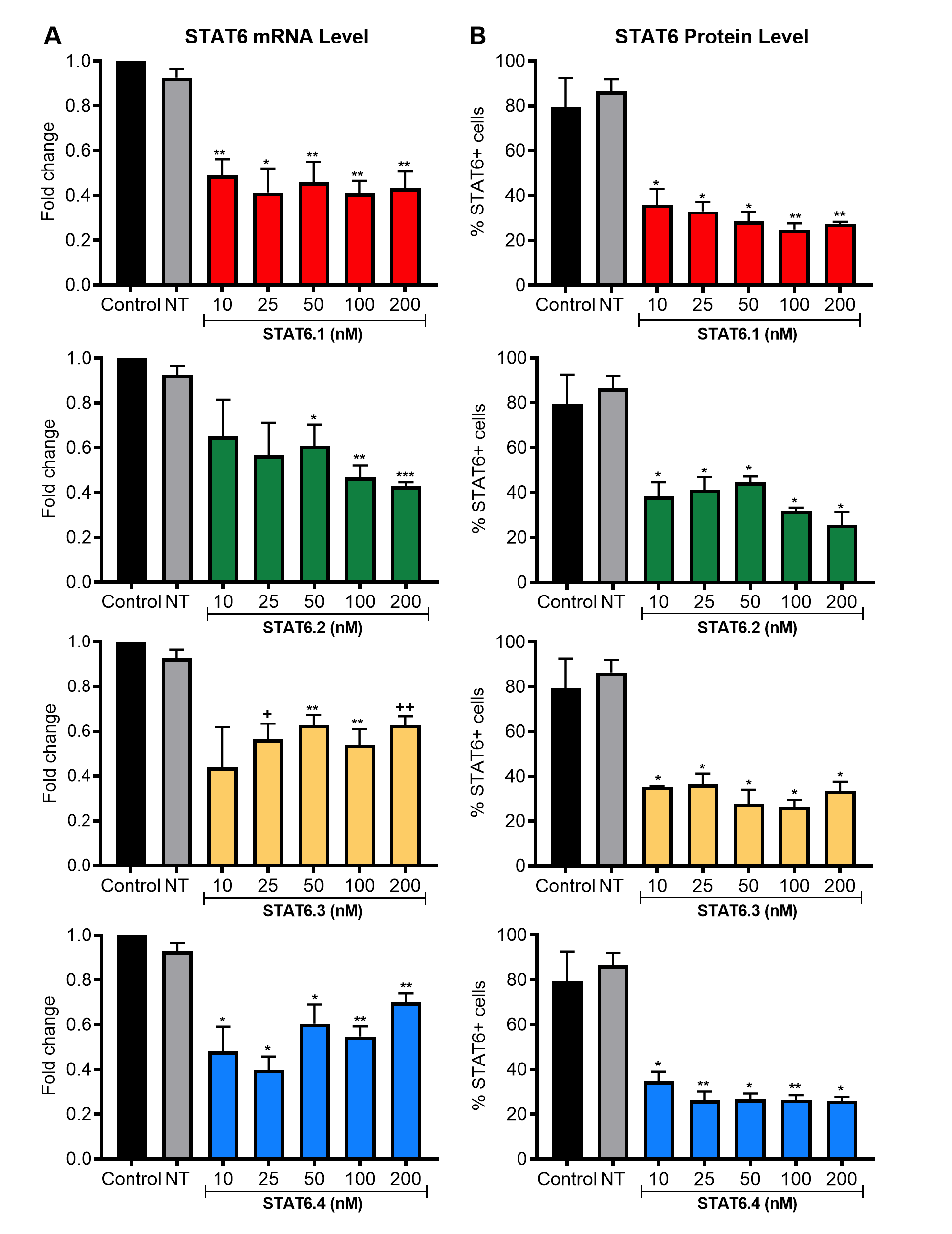

Supplement: S1 Fig — (A) STAT6 mRNA level measure at 24 hours post-transfection. The graphs represent the mean ± SEM of 3 inde- pendent experiments. Values were obtained by real-time PCR and results were analysed by ΔΔCt method for relative quantifications. The fold change is represented on the Y axes, and values are normalized to control cells. +: The p-value for a t-test is 0.0106, but, for non-parametric samples, p = 0.1 for an unpaired two-tailed Mann Whitney test. The significance of the result must be considered with caution. ++: The p-value for a t-test is 0.0056, but, for non-parametric samples, p = 0.1 for an unpaired two-tailed Mann Whitney test. The significance of the result must be considered with caution. (B) STAT6 protein level analysis. The graphs represent the mean of the percentage of STAT6 positive cells ± SEM of 2 independent experiments obtained by flow cytometry. The percentage of STAT6 positive cells is represented on the Y axes. STAT6 siRNAs and non-targeting siRNA were used at 10, 25, 50, 100 and 200 nM as the final concentrations. Control cells were non-transfected cells and STAT6 siRNA sequences 1, 2, 3 and 4 and non-targeting siRNA are denoted as STAT6.1, STAT6.2, STAT6.3 and STAT6.4 and NT, respectively. (TIF) [file pone.0246415.s003.tif]
