# Supplementary material for: Expression of Concern: STAT6 knockdown using multiple siRNA sequences inhibits proliferation and induces apoptosis of human colorectal and breast cancer cell lines
Source: PLoS One. 2021 Jan 28;16(1):e0246415. doi: 10.1371/journal.pone.0246415 (PMC7842988; doi:10.1371/journal.pone.0246415)
Supplement: S1 Table — (DOCX) [file pone.0246415.s004.docx]

| **Figure** | **p-value**  **(t test)** | **p-value**  **(Mann Whitney test)** | **Concordance** |
| --- | --- | --- | --- |
| **Fig1D**  **Day7 – NT* vs STAT6.1** | **0.002** | 0.1 | No  No significant differences when nonparametric test is applied |
| **Fig1D**  **Day7 – NT* vs STAT6.4** | **0.003** | 0.1 | No  No significant differences when nonparametric test is applied |
| S1_FigA  NT vs STAT6.3 25 nM | 0.0206 | 0.1 | No  No significant differences when nonparametric test is applied |
| S1_FigA  NT vs STAT6.3 200 nM | 0.0164 | 0.1 | No  No significant differences when nonparametric test is applied |

**S1 Table. List of data that did not pass normality test (****Shapiro-Wilk p-value<0.05).** Comparison of statistical significances obtained with parametric (t test) and non-parametric (Mann Whitney test) tests.
