# Supplementary material for: Expression of Concern: STAT6 knockdown using multiple siRNA sequences inhibits proliferation and induces apoptosis of human colorectal and breast cancer cell lines
Source: PLoS One. 2021 Jan 28;16(1):e0246415. doi: 10.1371/journal.pone.0246415 (PMC7842988; doi:10.1371/journal.pone.0246415)
Supplement: S2 Table — Comparison of statistical significances analyzed with equal (t test) and unequal (Welch´s correction) distribution variance tests. (DOCX) [file pone.0246415.s005.docx]

| **Figure** | **p-value**  **(t test)** | **p-value**  **(Welch´s correction)** | **Concordance** |
| --- | --- | --- | --- |
| Fig1B  Day 5 - NT vs STAT6.1 | 0.0019 | 0.0167 | Yes  Both significant |
| Fig2B  NT vs STAT6.1 | <0.0001 | <0.0001 | Yes  Both significant |
| Fig5A  Day2 - NT vs STAT6.1 | 0.0085 | 0.0393 | Yes  Both significant |
| Fig5G  NT vs STAT6.1 | = 0.3455 | 0.2001 | Yes  Both non-significant |

**S2 Table.** **List of data that did not pass equality of variance test (F test p-value<0.05)**. Comparison of statistical significances analyzed with equal (t test) and unequal (Welch´s correction) distribution variance tests.
