# Supplementary material for: Expression of Concern: STAT6 knockdown using multiple siRNA sequences inhibits proliferation and induces apoptosis of human colorectal and breast cancer cell lines
Source: PLoS One. 2021 Jan 28;16(1):e0246415. doi: 10.1371/journal.pone.0246415 (PMC7842988; doi:10.1371/journal.pone.0246415)
Supplement: S3 Table — (DOCX) [file pone.0246415.s006.docx]

**S3 Table.** **Resume of cases where n<3**. Comparison of statistical significances obtained assuming Gaussian distribution and equal variances (t test), non-parametric distribution (Mann Whitney test) and unequal variances (Welch´s correction test).

| **Figure** | **p-value**  **(t test)** | **p-value**  **(Mann Whitney test)** | **p-value**  **(Welch´s correction)** | **Concordance** |
| --- | --- | --- | --- | --- |
| Fig1D  Day 4 - NT vs STAT6.4 | 0.0015 | 0.1333 | 0.0801 | No  No significant differences when nonparametric distribution or unequal variances are considered* |
| Fig2D  NT vs STAT6.4 | 0.4112 | 0.8000 | 0.3413 | Yes  No significant differences found in any case |
| Fig3B  NT vs STAT6.1 (Early apoptosis) | 0.3432 | 0.8000 | 0.5052 | Yes  No significant differences found in any case |
| Fig3B  NT vs STAT6.1 (Late apoptosis) | 0.4776 | 0.8000 | 0.6226 | Yes  No significant differences found in any case |
| Fig3B  NT vs STAT6.1 (Total apoptosis) | 0.3924 | 0.8000 | 0.5735 | Yes  No significant differences found in any case |
| S1Fig_B STAT6.1  NT vs 10 nM  NT vs 25 nM  NT vs 50 nM  NT vs 100 nM  NT vs 200 nM | 0.0305  0.0166  0.0144  0.0099  0.0090 | 0.3333  0.3333  0.3333  0.3333  0.3333 | 0.0347  0.0200  0.0170  0.0257  **0.0521** | Only 200 nM condition (in bold) showed non-significant differences when unequal variances are assumed. When non parametric distribution is assumed changes are non-significant |
| S1Fig_B STAT6.2  NT vs 10 nM  NT vs 25 nM  NT vs 50 nM  NT vs 100 nM  NT vs 200 nM | 0.0289  0.0296  0.0209  0.0109  0.0170 | 0.3333  0.3333  0.3333  0.3333  0.3333 | 0.0298  0.0297  0.0458  **0.0519**  0.0171 | Only 100 nM condition (in bold) showed non-significant differences when unequal variances are assumed. When non parametric distribution is assumed changes are non-significant |
| S1Fig_B STAT6.3  NT vs 10 nM  NT vs 25 nM  NT vs 50 nM  NT vs 100 nM  NT vs 200 nM | 0.0117  0.0206  0.0194  0.0110  0.0164 | 0.3333  0.3333  0.3333  0.3333  0.3333 | **0.0678**  0.0220  0.0199  0.0232  0.0212 | Only 10 nM condition (in bold) showed non-significant differences when unequal variances are assumed. When non parametric distribution is assumed changes are non-significant |
| S1Fig_B STAT6.4  NT vs 10 nM  NT vs 25 nM  NT vs 50 nM  NT vs 100 nM  NT vs 200 nM | 0.0180  0.0018  0.0273  0.0030  0.0246 | 0.3333  0.3333  0.3333  0.3333  0.3333 | 0.0212  0.0171  0.0293  0.0378  0.0419 | All conditions showed significant differences when unequal variances are assumed. When non parametric distribution is assumed changes are non-significant |
| S2Fig_A  NT vs STAT6.1  NT vs STAT6.2  NT vs STAT6.3  NT vs STAT6.4 | 0.8475  0.5289  0.3958  0.5507 | 0.6667  0.6667  0.6667  0.6667 | 0.8474  0.5765  0.4178  0.5733 | Yes  No significant differences found in any case |
| S2Fig_B  NT vs STAT6.1  NT vs STAT6.2  NT vs STAT6.3  NT vs STAT6.4 | 0.1758  0.3023  0.3275  0.0977 | 0.3333  0.3333  0.6667  0.3333 | 0.1988  0.3317  0.3541  0.0983 | Yes  No significant differences found in any case |
| S2Fig_C  NT vs STAT6.1  NT vs STAT6.2  NT vs STAT6.3  NT vs STAT6.4 | 0.0597  0.4887  0.1453  0.0568 | 0.3333  0.6667  0.3333  0.3333 | 0.1522  0.5080  0.2360  0.0995 | Yes  No significant differences found in any case |

*******Kruskal-Wallis test by multiple comparisons** (nonparametric test for comparison of 3 or more groups):

| Control vs. NT | >0,9999 |
| --- | --- |
| Control vs. STAT6.1 | 0,0898 |
| Control vs. STAT6.4 | 0,0777 |
| **NT vs. STAT6.1** | ***0,0469** |
| **NT vs. STAT6.4** | ***0,0411** |
