# Supplementary material for: Expression of Concern: STAT6 knockdown using multiple siRNA sequences inhibits proliferation and induces apoptosis of human colorectal and breast cancer cell lines
Source: PLoS One. 2021 Jan 28;16(1):e0246415. doi: 10.1371/journal.pone.0246415 (PMC7842988; doi:10.1371/journal.pone.0246415)
Supplement: S5 Table — (DOCX) [file pone.0246415.s008.docx]

**S5 Table. Statistical analysis of Figure 3**.

| **Figure** | **Passed normality test?**  **Shapiro-Wilk test** | **Passed equal variance test? F test** | **Comments** |
| --- | --- | --- | --- |
| Fig3A  Early apoptosis  Late apoptosis  Total apoptosis | yes  yes  yes | yes  yes  yes | t test performed |
| Fig3B  Early apoptosis  Late apoptosis  Total apoptosis | STAT6.1 n too small (all the rest passed) | STAT6.1 n too small (all the rest passed) | See Supporting Table S3 |
